# Takayasu’s Arteritis in Pregnancy: A Rare Case Report

**DOI:** 10.7759/cureus.28815

**Published:** 2022-09-05

**Authors:** Mayur Raka, Aarshika Singh

**Affiliations:** 1 Obstetrics and Gynaecology, Jawaharlal Nehru Medical College, Datta Meghe Institute of Medical Sciences, Wardha, IND

**Keywords:** pregnancy, carotid doppler changes, pulseless disease, vasculitis, takayasu’s arteritis

## Abstract

The aorta and its main branches are affected by the uncommon systemic vasculitis known as Takayasu's arteritis (TA). The gradual onset of the disease and early, very generalised symptoms may factor in the large delay in diagnosis and treatment. This is an interesting case report of a patient with the rare condition of TA, who was unaware of her disease. Her ailment was diagnosed during antenatal examination, and this report speaks of her further imminent management without any adverse effects on maternal or foetal outcomes. To enhance maternal and foetal prognosis, gynaecologists, cardiologists, radiologists, and neurologists must work together collaboratively. Here is a case study of a woman who had TA and bravely defied the condition's whims to give birth without issue.

## Introduction

Pulseless disease/young female arteritis, often known as Takayasu's arteritis (TA), is an uncommon, chronic inflammatory large vessel vasculitis of unknown cause that affects women of childbearing age [1]. The Japanese ophthalmologists Mikito Takayasu and Onishi were the first to report it. According to reports, there are 13 occurrences per million people. Women of Asian descent are the most likely to experience it [2]. It typically affects the aorta and its branches, causing narrowing, and aneurysms of systemic and pulmonary arteries in the body [3]. Young female arteritis, commonly referred to as TA, is an uncommon and persistent inflammatory condition of the major arteries. Asian-born women of reproductive age mostly suffer from the condition [4]. Females during pregnancy are more prone to cardiovascular problems such as hypertension and heart disease. Despite the disease's detrimental effects on maternal and newborn health, research on how to treat it effectively is still lacking [5].

The progression of the disease is unaffected by pregnancy per se; however, the second and third trimesters have the peak incidence of the condition. Due to the possibility of problems such as hypertension, multiple organ dysfunction, and stenosis obstructing regional blood flow, such patients require specific treatment throughout the peripartum period, which can result in intrauterine growth restriction and birth weight below 2500 grams (low birth weight) in newborns. Patients usually conceive without being aware they have TA or having started particular therapy for it because diagnosis delays are relatively typical [6]. A difficult problem still exists in providing the best care for pregnant patients with this disease, especially given the global drive towards multicentric large vessel vasculitis research and the early introduction of a levitating pool of targeted drugs. An ideal maternal and foetal prognosis frequently requires an interdisciplinary collaboration of cardiologists, obstetricians, neurologists, rheumatologists, and cardiologists. Here, a case is described, and the literature reviewed in light of the small-scale studies on active TA in pregnancy that have been published so far anyway, particularly in low-income countries, to sensitise obstetricians on how to treat this rare, typical, and clinically important condition, and its fetomaternal outcome.

## Case presentation

**Figure 1:**
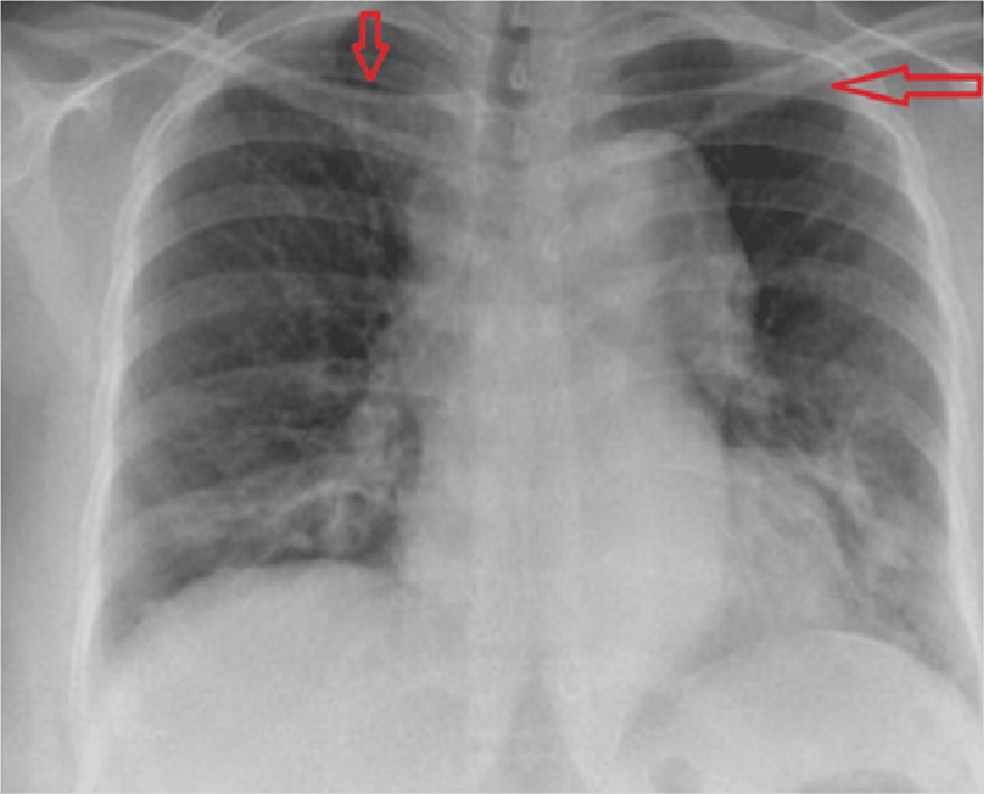
Chest X-ray showing widened mediastinum The red arrows show the widened mediastinum

A 21-year-old pregnant woman with primigravida with 8.4 weeks gestational age came with complaints of cold and sneezing for 15 days. All basic investigations were sent and were within normal limits except, on clinical examination, it was found that the recordings of blood pressure in the two arms were different. The right upper arm had a blood pressure of 96/60 mmHg, whereas the left upper arm had 102/60 mmHg; the left upper arm had more blood pressure. The radial pulse of the patient was feeble (pulselessness); the patient had no history of hypertension in the past and no relevant family history for TA. Bruit was appreciated very well in the right and left carotid and left radial arteries. Since the patient was a young Asian female with clinical features pointing towards a systemic vasculitis pathology, the attending started thinking along the lines of TA. Chest X-ray showed widened mediastinum, as shown in Figure 1, which is suggestive of vascular causes or aetiology.

Two-dimensional (2D) echocardiogram and carotid Doppler were advised. Figure 1 shows the 2D echocardiogram. Carotid artery Doppler was also done. Carotid Doppler of the right side is shown in Figure 3 and of the left side is shown in Figure 4. They are suggestive of hypoechoic diffuse intimal media wall thickening, causing luminal narrowing of bilateral common carotid arteries. The right subclavian showed diffuse intimal media wall thickening causing luminal narrowing. These are features suggestive of arteritis. However, bilateral internal carotid arteries were normal [7]. MRI was done for the aorta and major blood vessels but was not conclusive for any diagnostic value. Since is an autoimmune condition, we did a workup for antinuclear antibodies. For further investigation, the patient was not willing due to the high cost. A cardiology opinion was taken, and a diagnosis of the same was made. The patient was started on aspirin 75mg once a day (continued up until term and was discontinued two days before labour induction) and corticosteroids (40mg tapered to 20mg at term) for the same duration. Regular monitoring of all vital parameters like blood sugars, blood pressure, bleeding abnormalities, and most importantly, foetal growth and development was done throughout the pregnancy. Regular renal artery Doppler was also done.

**Figure 2:**
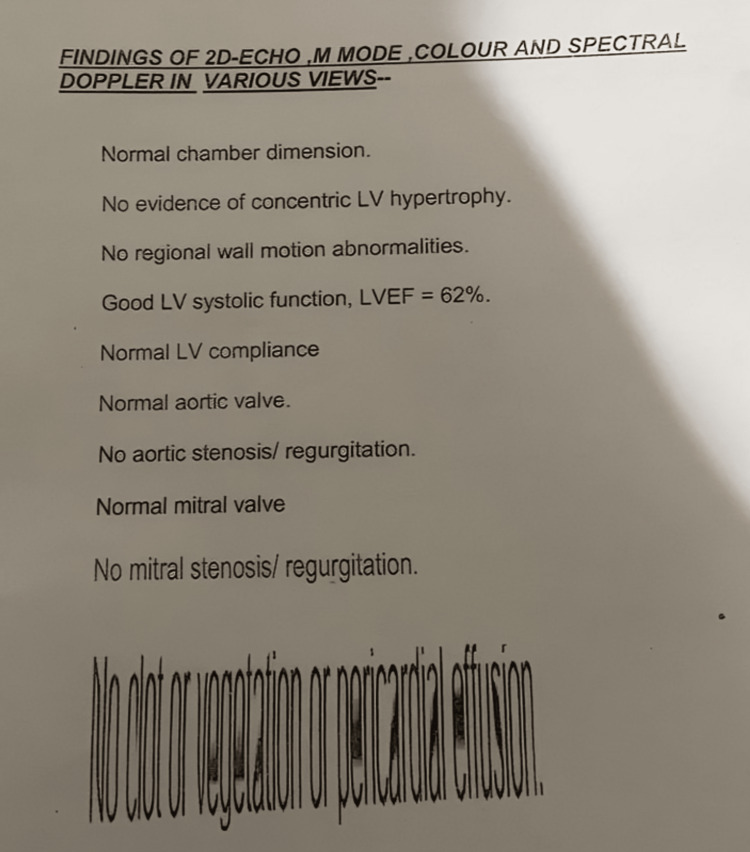
2D echocardiogram

**Figure 3:**
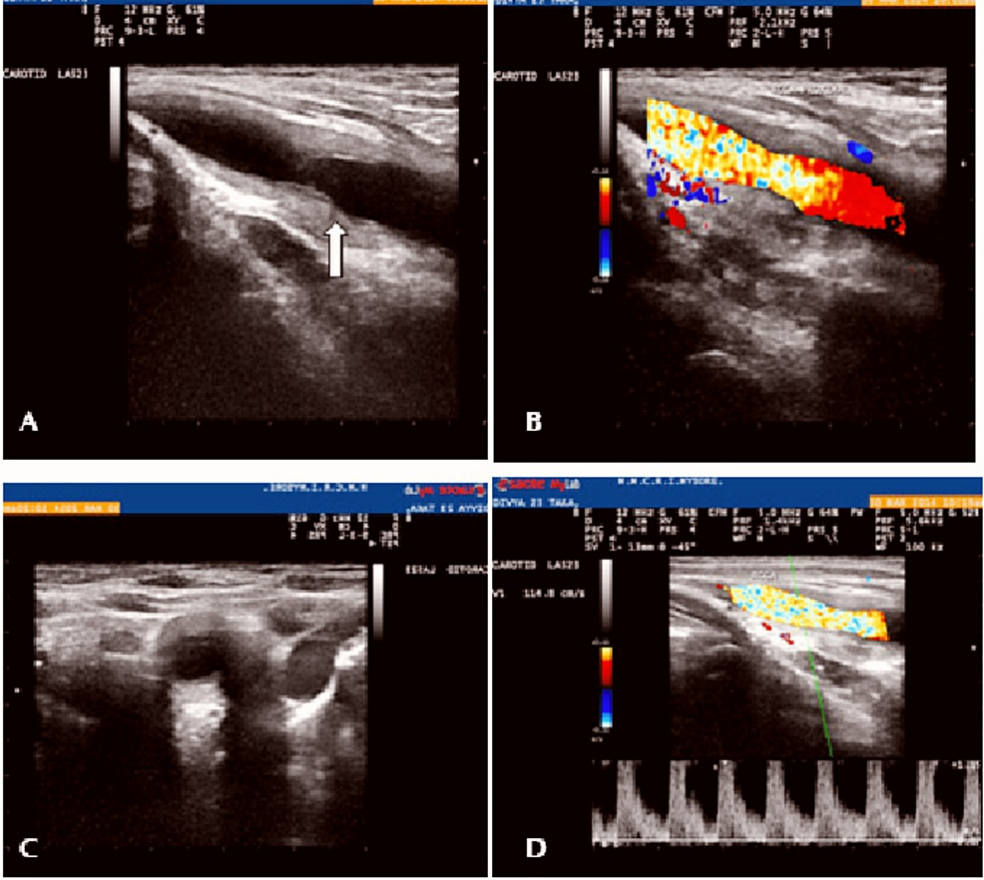
Right common carotid artery Doppler Long segment (shown by white arrow) diffuse homogenous wall thickening, spectral broadening of waveform in common carotid artery (CCA)

**Figure 4:**
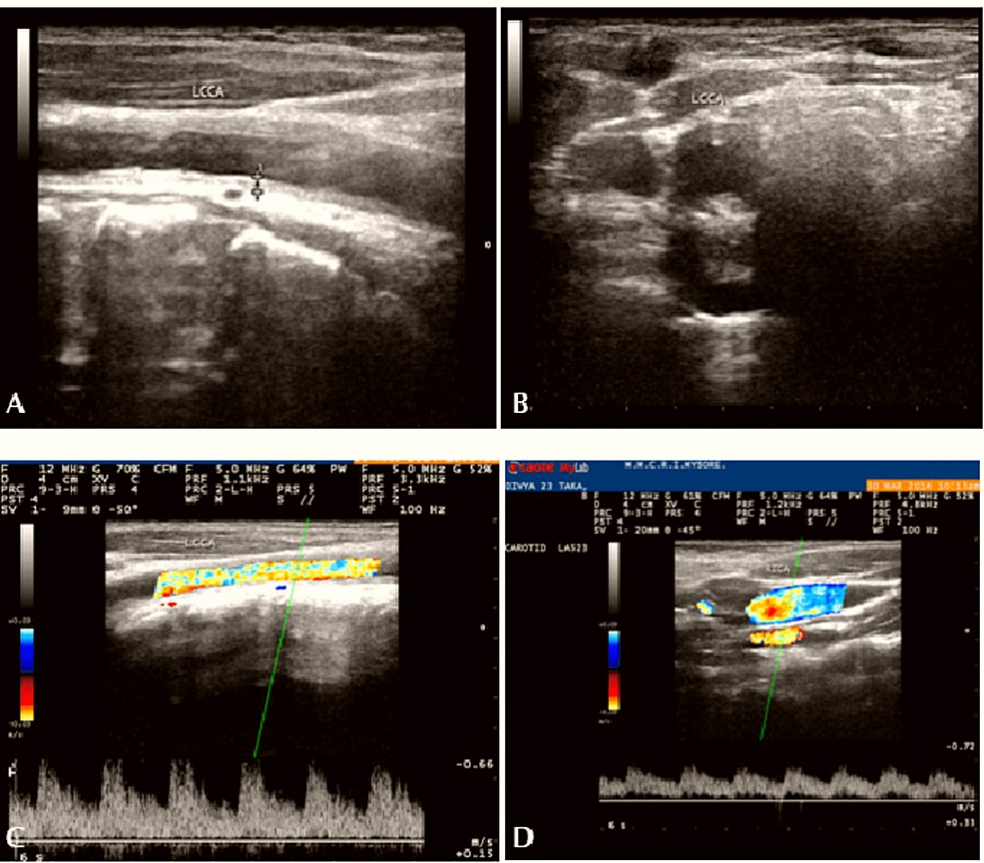
Left common carotid artery Doppler Carotid artery Doppler showing luminal narrowing in left carotid artery

As term approached, after consultation with the treating cardiologists, aspirin and corticosteroids were discontinued, and induction of normal labour was initiated. Mechanical dilatation and prostaglandins were the methods of choice. Physician's opinion on intrapartum management of labour was also sought. After strict fetal heart sound monitoring and blood pressure, pulse, peripheral oxygen saturation (SpO2), and bleeding per vagina monitoring, the patient was managed. The risks versus benefits of epidural analgesia were also discussed with the patient. However, at 37.6 weeks of gestational age, owing to non-progress of labour, the patient was taken for emergency caesarean section and a 2.8-kilogram female baby was delivered. The intra and postoperative periods were uneventful with close surveillance [8]. Post delivery, she was given injection of low molecular weight heparin (LMWH) for 36 hours and then later started on aspirin (Ecosprin) on the advice of attending cardiologist. Prednisone was continued. Contraceptive counselling was done in detail. When the patient came for follow-up post delivery, no abnormalities were found on local and systemic examination and lab workup. The patient was counselled for three monthly follow-ups regularly regarding her condition and pediatric follow-ups for the baby for regular normal vaccination and health check-up, but in this case report, it was not considered, because it was beyond our scope.

## Discussion

Women account for approximately 80% to 90% of TA cases, and the disease often manifests between the ages of 10 to 40 years [9]. The condition is prevalent worldwide, with Asia having the highest frequency [10]. Japan estimated roughly 150 new TA cases a year [13]. The aorta, branches, and pulmonary arteries are all affected by the chronic inflammatory condition known as TA. The inflammation results in varying degrees of stenosis, occlusion, or dilatation of the afflicted arteries. Since Mikito Takayasu, a Japanese ophthalmologist, officially reported the disease in 1908 [11], a great deal has been learnt about the condition, even if the actual cause and pathophysiology are still unclear. Furthermore, the symptoms might range from fever, ineptitude, and weight loss to potentially fatal hyponatremia, hypoglycemia, and cardiac failure. Obstetricians, cardiologists, rheumatologists, anesthesiologists, and neonatologists are among the specialists who work together to treat TA. The ultimate goals include reducing inflammation and treating critical concerns such as occlusive or stenotic lesions and hypertension [12].

Preconception treatment is essential for treating women of childbearing age having TA. However, such type of therapy will mainly concentrate on dosage adjustments, stopping cytotoxic medications, folic acid supplementation throughout the pre-conception phase, and the best time to become pregnant. Similarly, patients are recommended to plan an early visit for routine prenatal care because TA is possible throughout the remission phase; pregnancy should also be planned for better outcomes without much problem. In addition to standard antenatal checkups, serial monitoring of blood pressure, renal function, and cardiac status, and pre-eclamptic screening are critical in these patients. The daily count of foetal kicks, gravidogram, serial foetal biometry, biophysical profile, and foetal Doppler are also essential components of foetal surveillance. Pregnancy does not impact TA's progress. However, maintaining blood pressure control is crucial since any rise could cause an aneurysm to burst, causing hypotension or maternal cerebral ischemia. The ineffectiveness of peripheral blood pressure monitoring may also make it more challenging to treat hypertension in these people because upper extremity pulses may be absent. The monitoring of blood pressure several times in extremities or invasive blood pressure monitoring can benefit patients.

However, managing blood pressure may be challenging because of the physiological changes that occur during pregnancy. Any patient with TA should make plans to get pregnant once their blood pressure and condition are stable. Antihypertensive medicine must be regulated, and angiotensin inhibitors and angiotensin-converting enzyme (ACE) inhibitors must be contraindicated. On the other hand, it has been demonstrated that hypertension which is out of control during pregnancy is associated with aortic dissection, cardiac and renal failure, stroke, and maternal death. Ultimately, for patients with TA, vaginal delivery has emerged as the optimal method of labour management. Epidural analgesia has also been promoted for use during childbirth.

## Conclusions

This is an exciting report of successful pregnancy and live birth after the diagnosis of TA in a pregnant female, who despite the cardiovascular risk was safely delivered with a healthy child. TA being a rare disease, not a lot has been studied about its correlation with pregnancy. Symptomatic treatment remains the mainstay of treatment for now. Obstetricians face a difficult problem while caring for pregnant women with TA. The ideal care for pregnant individuals with this condition remains elusive despite improvements in cardiovascular management and the introduction of cutting-edge medications. Further studies are required to further our understanding of disease progression.
